# Acute Duodenal Ulcer Perforation Following Tirzepatide Treatment: A Case Report

**DOI:** 10.7759/cureus.80671

**Published:** 2025-03-16

**Authors:** Maho Hayashi, Koji Hayashi, Yusuke Tsujigiwa, Seiko Uwafuji, Mamiko Sato, Yasutaka Kobayashi

**Affiliations:** 1 Department of Internal Medicine, Fukui General Hospital, Fukui, JPN; 2 Department of Rehabilitation Medicine, Fukui General Hospital, Fukui, JPN; 3 Department of Gastroenterology, Fukui General Hospital, Fukui, JPN; 4 Graduate School of Health Science, Fukui Health Science University, Fukui, JPN

**Keywords:** drug-induced side effects, duodenal perforation, duodenal ulcers, gastroduodenal ulcer, helicobacter pylori, side-effects, “tirzepatide”

## Abstract

We present the case of a 36-year-old man with a history of type 2 diabetes mellitus (T2DM) and prior plantar necrotizing fasciitis, who initially improved on semaglutide but later experienced weight gain and elevated hemoglobin A1C (HbA1c) levels. After switching to tirzepatide, he developed nausea, epigastric pain, and vomiting. Shortly after increasing the tirzepatide dosage, he presented to the emergency room with severe abdominal pain. Diagnostic imaging revealed an ulcer in the duodenal bulb with free air, indicating acute duodenal ulcer perforation. The patient underwent emergency surgery, and postoperative tests confirmed a *Helicobacter pylori *infection. He received eradication therapy and was discharged about two months later.

This case highlights the potential risk of duodenal perforation associated with tirzepatide, particularly in patients with untreated* Helicobacter pylori *infection. The patient's worsening gastrointestinal symptoms and perforation after initiating tirzepatide suggest a potential drug-related effect, emphasizing the need for careful monitoring and further research into the mechanisms underlying gastrointestinal complications associated with tirzepatide.

## Introduction

Tirzepatide is a new medication for type 2 diabetes mellitus (T2DM) that helps manage blood sugar levels and supports weight loss [1]. Recent research has indicated that long-acting glucagon-like peptide-1 (GLP-1) receptor agonists, such as semaglutide and liraglutide, as well as sodium-glucose cotransporter-2 (SGLT2) inhibitors like empagliflozin, are effective strategies for both weight management and blood sugar control in patients with obesity and related conditions [2]. In contrast, tirzepatide functions as a dual agonist that targets both glucagon-like peptide-1 (GLP-1) and glucose-dependent insulinotropic polypeptide (GIP) receptors, potentially offering superior weight loss efficacy compared to these other options [2]. This advantage is due to its distinctive mechanism of action, which harnesses the combined effects of both GLP-1 and GIP [2]. Therefore, tirzepatide emerges as a highly promising candidate in the continuum of treatments for obesity and type 2 diabetes [2]. Additionally, tirzepatide may reduce the risk of cardiovascular diseases, likely owing to its effects on weight loss, blood sugar control, and inflammation [1,3].

While tirzepatide may cause gastrointestinal side effects such as nausea, vomiting, and diarrhea [1,2], there have been no reports of gastroduodenal ulcers. This case report suggests a potential association between tirzepatide and acute duodenal ulcer perforation.

## Case presentation

A 36-year-old man with a history of type 2 diabetes mellitus (T2DM) since age 26 and plantar necrotizing fasciitis at 28 began treatment with semaglutide alongside oral medications, including metformin, canagliflozin, vildagliptin, and rosuvastatin. He had no documented history of using non-steroidal anti-inflammatory drugs (NSAIDs) or ulcer disease. His body mass index was 30.2 (height: 190 cm, weight: 109 kg), and his serum hemoglobin A1c (HbA1c) level was 7.5%. He had a family history of T2DM in both his parents and his maternal grandmother. After starting semaglutide, his HbA1c improved to 6.0-6.3%, and he lost approximately 2 kg over one year. At age 38, he gained weight, and his HbA1c rose to 7.0%, prompting a switch from semaglutide 1.0 mg to tirzepatide 2.5 mg weekly, which caused nausea. Clinical trials had reported nausea as a common side effect during dose escalation with tirzepatide, but it was often transient [2]. Thus, the decision was made to continue tirzepatide for this patient. 

**Figure 1:**
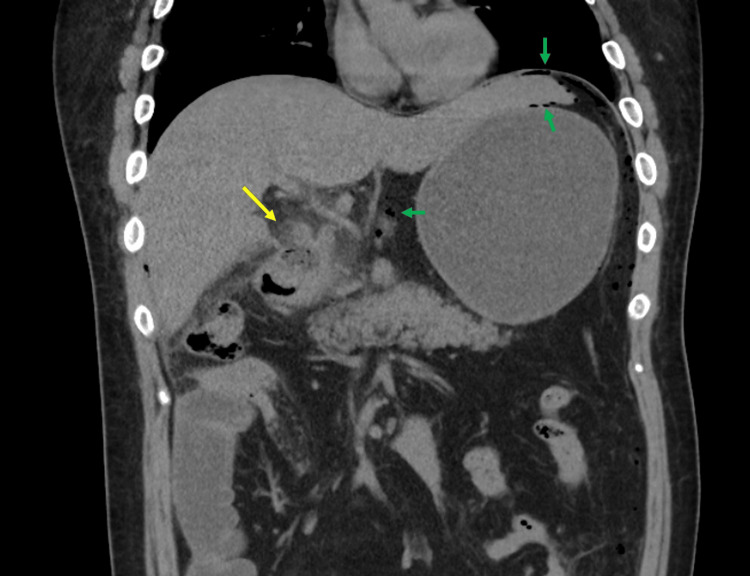
Abdominal computed tomography (CT) imaging. Abdominal CT shows a rupture of the intestinal wall and an increase in the CT value of the surrounding adipose tissue in the posterior upper wall of the duodenal bulb, suggesting a perforated duodenal ulcer (yellow arrow). Furthermore, free air is observed throughout the peritoneal cavity (green arrows).

Four weeks later, the tirzepatide dose was increased to 5.0 mg weekly, leading to nausea and occasional epigastric pain. Two weeks after the dose increase, he experienced vomiting and severe epigastric pain followed by severe pain in his right flank, prompting a visit to our emergency room. Vital signs showed a temperature of 35.9°C, blood pressure of 95/49 mmHg, and a pulse rate of 105 bpm. Abdominal examination revealed a flat, soft abdomen with tenderness and rebound tenderness in the epigastrium. Blood tests indicated elevated aspartate aminotransferase, alanine aminotransferase, and C-reactive protein levels. An abdominal CT scan revealed an ulcer in the posterior-superior wall of the duodenal bulb and free air in the abdominal cavity (Figure 1).

He was diagnosed with acute duodenal ulcer perforation and underwent emergency surgery with laparoscopy. Postoperatively, *Helicobacter pylori* (*H. pylori*) antibodies tested positive. He received antibiotic therapy, including *H. pylori* eradication therapy, and was discharged approximately two months later.

## Discussion

We present a case of acute duodenal ulcer perforation that occurred after switching the medication from semaglutide to tirzepatide. The patient had an untreated *Helicobacter pylori *infection. He did not exhibit gastrointestinal symptoms while on semaglutide; however, he experienced nausea after starting tirzepatide. The perforation was noted while the dosage of tirzepatide was increased to 5.0 mg weekly.

Exogenous GLP-1 effectively lowers glucose in critically ill patients on enteral feeding by stimulating insulin secretion and slowing gastric emptying, which may increase gastric acid exposure and impact the mucosa of the stomach and duodenum [4]. In contrast, GIP primarily enhances glucose-dependent insulin secretion and reduces gastrin and related acid secretion from gastric parietal cells [5]. GIP/GLP-1 dual agonists are believed to reduce gastrointestinal symptoms compared to GLP-1 alone; however, nausea and vomiting persist in some patients [6]. This may result from suboptimal dosing in clinical trials or differences in the pharmacodynamic profiles of single-molecule dual agonists versus their individual components [6].

Here, the patient had one of the well-established risk factors for gastroduodenal ulcers of *H. pylori *infection. While switching from semaglutide (a GLP-1 agonist) to tirzepatide (a GIP/GLP-1 co-agonist) worsened symptoms, including nausea and epigastric pain, ultimately leading to acute duodenal ulcer perforation, exact contributing factors are unclear. Although the ulcer could be attributed to *H. pylori *alone, the close temporal relationship between the onset of the ulcer and the increased tirzepatide dose, a medication known for gastrointestinal side effects, suggests a possible role for the drug. Alternatively, the combination of *H. pylori* infection and tirzepatide may have synergistically increased the risk of ulcers. Further research is needed to investigate the specific mechanisms behind gastroduodenal perforation associated with tirzepatide, particularly in the context of pre-existing *H. pylori* infection.

## Conclusions

This case highlights the potential risk of duodenal perforation associated with the use of tirzepatide, particularly in patients with untreated* Helicobacter pylori* infections. While the dual agonist combines the properties of GLP-1 and GIP, leading to improved glycemic control, it may also exacerbate gastrointestinal issues such as nausea and epigastric pain. The timing of the perforation following an increased dose of tirzepatide suggests a possible drug-related effect, especially given its known gastrointestinal side effects. Furthermore, the coexistence of *Helicobacter pylori* may synergistically elevate the risk of gastroduodenal ulcers. These findings underscore the need for careful monitoring and further investigation into the mechanisms underlying gastrointestinal complications associated with tirzepatide, particularly in patients with pre-existing risk factors.
